# Occupational Heat Stress and Kidney Health in Salt Pan Workers

**DOI:** 10.1016/j.ekir.2023.04.011

**Published:** 2023-04-20

**Authors:** Vidhya Venugopal, Robin Lennqvist, PK Latha, Rekha Shanmugam, Manikandan Krishnamoorthy, Nandhini Selvaraj, Rajagurusamy Balakrishnan, R. Omprashant, Anil Jacob Purty, Joy Bazroy, Jason Glaser, Kristina Jakobsson

**Affiliations:** 1 Department of Environmental Health Engineering, Sri Ramachandra Institute of Higher Education and Research, Chennai, India; 2 School of Public Health and Community Medicine, Institute of Medicine, Sahlgrenska Academy, University of Gothenburg, Gothenburg, Sweden; 3 Department of Community Medicine, Pondicherry Institute of Medical Sciences, Puducherry, India; 4 La Isla Network, Washington, USA; 5 Department of Occupational and Environmental Medicine, Sahlgrenska University Hospital, Gothenburg, Sweden

**Keywords:** heat strain, occupational heat stress, reduced kidney function, salt pan, WBGT

## Abstract

**Introduction:**

Work in heat affects millions of workers. Although kidney function in agricultural workers is increasingly researched, nonagricultural studies are scarce. In coastal salt pans, the absence of occupational exposures to pesticides and other toxicants allows assessment of heat stress alone.

**Methods:**

Seven Indian salt pans were surveyed from 2017 to 2020. Job-specific workload was assessed. Heat stress was characterized as exceeding the wet bulb globe temperature (WBGT)-threshold limit value (TLV) for high and moderate workloads. Preshift and postshift heart rates (HRs), tympanic temperatures, and urine specific gravity (USG) were measured for 352 workers, as were sweat rates (SwR), serum creatinine (SCr), serum uric acid, and urine dipstick. Estimated glomerular filtration rate (eGFR; ml/min per 1.73 m^2^) was computed. Heat-strain symptoms were assessed using questionnaires.

**Results:**

The mean WBGT was 30.5 ± 1.3 °C (summer) and 27.8 ± 1.9 °C (winter). Water intake during the workday was low, median was one Litre, and most workers (87%) exceeded the TLV for heat stress. Dehydration-related symptoms were frequent in those with high-heat stress, as were cross-shift increases in temperature (≥1°C; 15%), a high USG (≥1.020; 28%), and a high SwR (≥1 l/h; 53%). An eGFR of 60 to 89 ml/min per 1.73 m^2^ was observed in 41% of all workers examined, and 7% had eGFR below 60 ml/min per 1.73 m^2^. The odds ratio for eGFR <90 ml/min per 1.73 m^2^ in workers exceeding the TLV, compared to workers below this limit, adjusted for age and gender was 2.9 (95% CI: 1.3−6.4).

**Conclusion:**

Workplace interventions to prevent heat stress and dehydration in the salt pans and other at-risk industries are urgently required. The findings strengthen the notion that high-heat stress and limited hydration is a risk factor for kidney dysfunction.

## Graphical abstract

**Figure:**
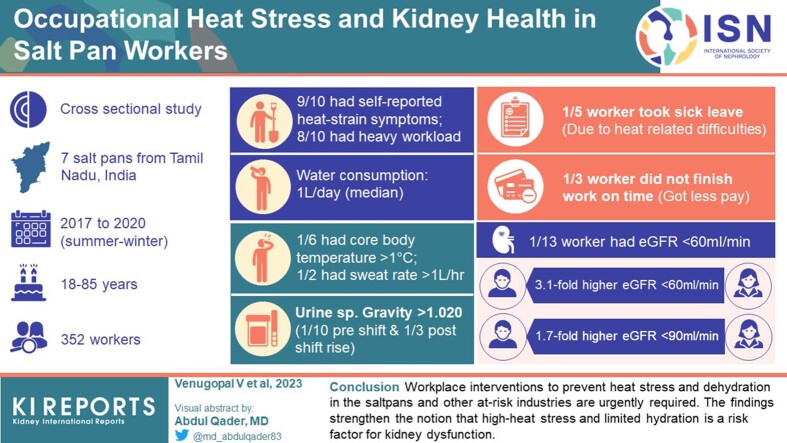

See Commentary on Page 1283

Climate change is expected to increase temperatures by at least 1.5 °C around the globe in the coming decades, with an increasing frequency and intensity of heat waves already here.^1^ Climate change has detrimental health, wealth, and economic repercussions, especially in low-income countries. Increased heat will have a considerable impact on the working population, particularly manual laborers in outdoor settings.^2^^,^^3^ According to the Global Climate Risk Index (2020),^4^ India is one of the most vulnerable regions to climate change, with projections of large economic losses because of heat-related illnesses and deaths.^5^, ^6^, ^7^ Several studies have shown that heat stress causes health problems among Indian workers.^8^, ^9^, ^10^, ^11^, ^12^, ^13^

Heat stress is defined as the net load resulting from the combined contributions of environmental heat, metabolic heat, and clothing.^14^ The role of metabolic heat generation during manual labor in hot climates is increasingly recognized.^15^ Reduced renal function, acute kidney injury, and urolithiasis have all been linked to heat stress from repeated and sustained vigorous exertion in hot environments, with or without dehydration.^16^, ^17^, ^18^ Heat stress and dehydration are thought to be major driving forces of chronic kidney disease of undetermined causes (CKDu), a form of kidney disease not caused by diabetes, hypertension, or other well established etiology; however, other risk factors may also be present. CKDu is an emerging noncommunicable disease, recognized among manual laborers in tropical countries around the globe.^18^, ^19^, ^20^, ^21^, ^22^

The majority of epidemiologic research on CKDu has hitherto been done in agricultural settings, in line with the alternative theory that pesticides, agrichemicals, or other environmental toxins are the main culprits.^23^ Chronic interstitial nephritis in agricultural communities has been recommended as a descriptive name for the outbreak of CKDu in Sri Lanka.^24^ Therefore, to have a better understanding of the role of heat stress in CKDu, researchers must look into laborers carrying out heavy work in other high environmental temperatures. One such opportunity is the salt industry.

In many coastal areas around the world, salt pan work is a common occupation largely done in the informal sector where the workers do not have fixed employment terms and workplaces are not formally registered.^25^ In and around Tamil Nadu, India’s southwest region, there is an estimate of 50,000 salt workers.^26^ The climate is hot and humid during the salt harvest season.^27^ Because of intense sunlight and reflections on white salt, sand, and flat water surfaces, ultraviolet exposures are exceptionally high.^28^ Heat, high perspiration rates, high humidity, and salt irritate the skin and eyes, increasing the risk of dermatologic and ophthalmologic issues.^28^ The few studies that have been published about salt pan workers have largely focused on hypertension, ophthalmologic, and cutaneous problems.^29^^,^^30^ However, a recent Thai study reported decreased eGFR in salt pan workers with a high workload and dehydration over a harvest season.^31^ In light of this, the aim of our research was to describe heat stress, heat-related symptoms, and kidney function among salt pan workers in Tamil Nadu, India.

## Methods

### Setting and Study Population

**Figure 1:**
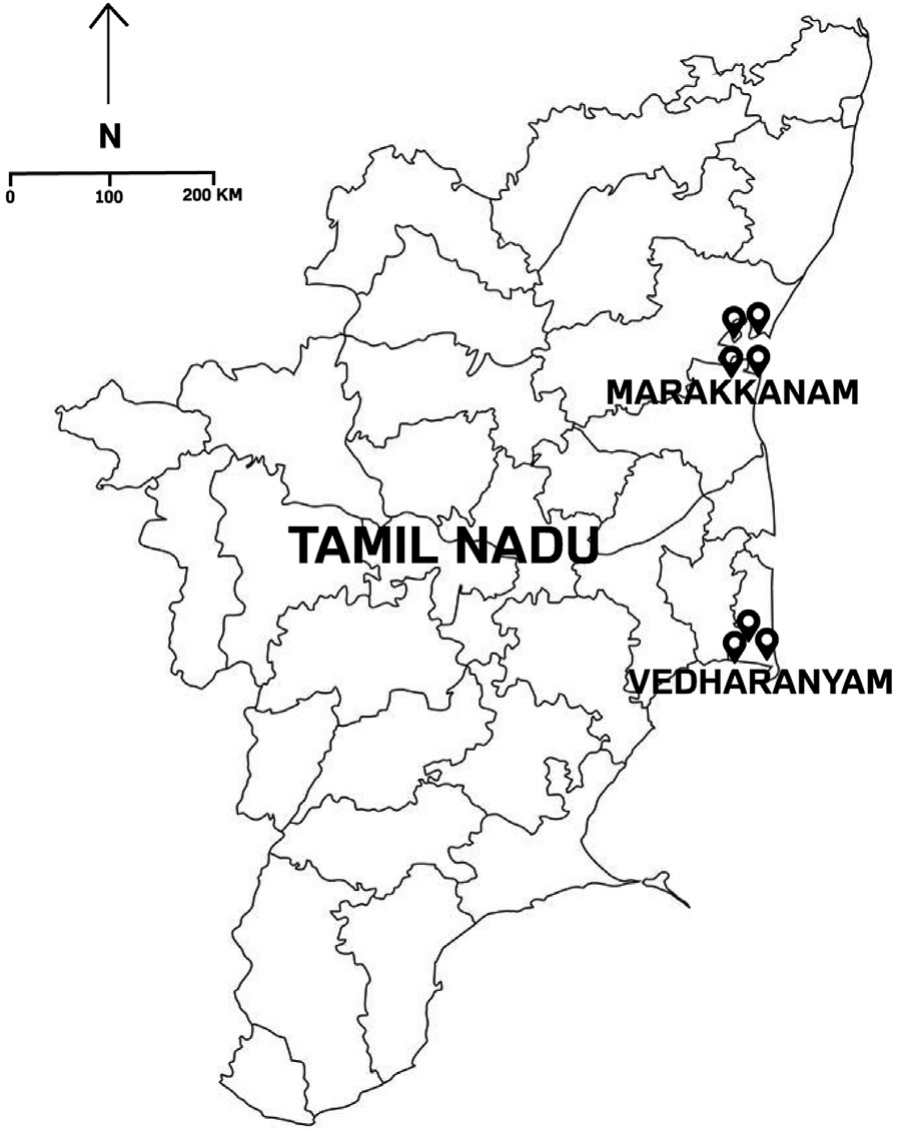
Study locations of salt pans in Tamil Nadu, India

We conducted a cross-sectional investigation among salt pan workers. After initial contacts and walk-through surveys by a certified industrial hygienist (author VV), we chose 7 salt pans, 4 in Marakkanam and 3 in Vedharanyam, Tamil Nadu, India (Figure 1). The study was conducted during the summer (April–July) and winter (October–January) seasons between 2017 and 2020, thus capturing the seasonal changes.

Work tasks in the salt pans included mud-border making, trampling the ground, salt crystal reshuffling, salt-scrapping, raw salt heaping, loading, packing, crushing, mill operations, and supervision. Working hours were between 6 am and 2 pm, with a lunch break of 30 minutes to 1 hour in the open air. The workers did not have any other breaks during active working hours. Apart from sparse vegetation, there were no shaded areas for the workers to rest. The workers brought water bottles from home and could refill them at the workplace, and the sanitation was poor with no toilet facilities. Whereas the salt pans in Vedharanyam were not run in an industrial way, the salt pans in Marakkanam were run in a partly industrial way with stricter production goals and piece rate wages. The laborers were largely locals who work in the salt pans on a seasonal basis. During the half-year long rainy period when the salt pan is idle, the workers hunt for other informal work.

On the days of workplace visits, we aimed to recruit workers who had more than 1 year of working experience in the salt pans. By this convenience sampling, approximately 20% of the daily workforce was included. Of the 380 salt pan workers who were initially recruited, 9% reported preexisting known medical disorders such as diabetes or hypertension and were therefore excluded, leaving a final sample group of 352 workers. We investigated 201 workers in the summers of 2017, 2018, and 2019; and 56 workers in the winter of 2018 in the salt pans in Vedharanyam. In Marakkanam, we investigated 53 workers during the summer of 2018, and 42 workers during the winter of 2020. All workers were acclimatized to the environmental conditions. The Institutional Ethics Committee at Sri Ramachandra Institute of Higher Education and Research (IEC-NI/17/APR/59/54) approved the study, and the workers gave their informed consent to participate.

### Questionnaires

Trained field workers administered a questionnaire translated from the High Occupational Temperature Health and Productivity Suppression questionnaire.^32^ The questions were translated into Tamil by SR, PK, and KM, and further developed with the help of local Tamil expertise so as to make the questions understandable for the workers. After piloting in the field, we made modifications again as per the suggestions of the workers for easy understanding of questions. The questions were read to the participants and registered by the field workers. The questionnaire had various sections which include demographic information, occupational heat exposure (work duration, timing, and years of exposure), socioeconomic characteristics, chemical and pesticide exposures, infections, snake bites, diabetes, hypertension, symptoms of heat-related illnesses, coping mechanisms, productivity losses (absenteeism, sick leave because of heat, wages lost), access to welfare facilities, menstrual history (for women), and past medical history regarding any chronic diseases.^10^

Using a structured interview, we gathered information about job history, socioeconomic characteristics, chemical and pesticide exposures, infections, snake bites, diabetes, hypertension, and heat stroke. Heat stress and heat strain symptoms such as excessive sweating, thirst, tiredness, cramps, headache, nausea, vomiting, fainting, or prickly heat/rashes, as well as urogenital issues such as burning sensations, rashes, and urinary tract infections were collected using a modified version of the “High Occupational Temperature Health and Productivity Suppression” questionnaire^32^ in English, which was translated into Tamil during the interview. The survey's questions about heat-related illnesses referred to the ongoing season.

### Assessment of Environmental Heat and Heat Stress Exposure

We measured environmental climatic conditions according to the American Conference of Governmental Industrial Hygienists 2018 guidelines, using a calibrated portable heat stress WBGT monitor (QuesTemp°34; QUEST Technologies, USA) with an accuracy level of 0.5 °C between 0 °C and 120 °C of dry bulb temperature and 5% relative humidity.^33^ In total, 100 measurements were taken during 19 days in both summer and winter conditions in Marakkanam (June and January), and Vedharanyam (April to July and October). We measured WBGT at all the work sites using repeated periodic measurements (15 minutes each) on monitoring days throughout the course of a shift (6 am to 2 pm). Assessment for each specific work task was done in about 2 to 3 shifts, totaling 8 measurements across the 2 seasons. To determine the workers' attributed WBGT exposure during a work shift, we monitored the hourly ambient WBGT during normal shift hours and averaged the hourly data. To assess the risk of heat stress on the day of investigation (above or below TLV-WBGT), we categorized the workers using the corresponding attributed WBGT with the American Conference of Governmental Industrial Hygienists TLV of 27.5 °C for heavy workload and 28 °C for moderate workload^34^ settings that would require rest periods if exceeded.

Experienced occupational hygienists (author VV) evaluated workloads while taking WBGT measures and classified each job type following the American Conference of Governmental Industrial Hygienists screening limits for work category.^17^^,^^33^ All jobs were categorized as either moderate workload (trampling the ground, leveling it with river sand, and constructing the pan walls from mud) or heavy workload (salt crystals raked together, collected in piles, moved to bigger piles, loaded in sacks, transported in 25 kg sacks, and stacked on top of salt piles); none were categorized as light workload.^33^

### Physiological Measurements

Each worker was examined twice, first in the morning before the start of work, then again during and at the end of the workday. The tympanic temperature, a proxy for core body temperature, was measured using an infrared ear thermometer (BRAUN ThermoScan IRT 6020). A temperature increase of more than 1 °C during the workday was deemed unfavorable.^35^

Workers’ preshift weight was assessed once they reached the field, using a digital weighing scale (Dr. Gene, model no: MS-7703). Midshift, usually after 2 to 5 hours, each participant’s weight was assessed before delivering their urine samples, and fluid consumption was reported. Thereafter, a urine spot sample was obtained. SwR was calculated by dividing the difference in preshift and midshift body weight plus self-reported fluid consumption by the observation time.^35^ A SwR of ≥1 l/h was considered as high.^35^ Urinating throughout the observed period was infrequent, particularly among women, because of the lack of toilet facilities at the workplace. According to our observations, females usually waited 5 to 6 hours before going in search of a urinating spot. HR was measured during approximately 1 minute using a Polar M430 bluetooth watch before the start of the workday and during the break or at the end of the workday, immediately after the completion of work. An HR of over 100 beats per minute was seen as excessive.^10^^,^^11^^,^^33^^,^^36^

Two different kinds of urine dipsticks (Dirui H10 and Siemens Multistix) were used to assess proteinuria, hematuria, pH, and leucocytes. The USG was determined using a refractometer, with USG ≥1.020 being considered excessive.^37^, ^38^, ^39^ Venous blood samples were taken at any time during the workday that was convenient for the workers. Unfortunately, field logistics did not allow venipuncture before the work shift, which would have been preferrable for a baseline assessment of S-Cr and eGFR. At a nearby authorized laboratory, the traditional Jaffe method was employed to determine S-Cr.^40^ The eGFR was calculated using the Chronic Kidney Disease Epidemiology Collaboration equation.^41^

### Data Analysis

We used Microsoft Excel for data entry and consolidation. For demographic variables, heat exposure (WBGT °C), self-reported heat-related health symptoms, and physiological indicators, descriptive statistics were utilized. The dependent variables (heat strain symptoms, symptoms of dehydration, urinogenital issues, CBT, SwR, USG, and eGFR) were categorized according to the standard range. Chi-square tests were used to examine categorical variables. The Wilcoxon signed rank test was used to analyze comparisons between preshift and postshift physiological heat strain markers because it is continuous data. Multivariate logistic regression models employing a stepwise strategy for controlling probable confounders yielded adjusted odds ratios with a *P*-value cut off of 0.05. To begin computing multivariate logistic regression, we performed the chi-square test to determine the significance of the independent variable in relation to covariates. The second stage involved calculating the adjusted odds ratios between the independent and dependent variables after controlling for major confounding variables (gender and age). In the second stage, the other nonsignificant confounders were excluded. Considering that the number of years of exposure is dependent on age, a correlation analysis was conducted. A Spearman correlation coefficient test shows age and years of exposure were moderately correlated (0.6); therefore, age (categorized) was used as one of the confounders in the main analysis. The multivariate logistic regression was not adjusted for smoking or alcohol intake, which exclusively occurred in men; however, correcting for gender eliminates this confounding factor. We utilized SPSS statistical program version 16.1 (IBM Corporation, New York, USA) and Microsoft Excel for data analysis and for preparing the graphs.

## Results

### Study Population and Demographics

The median age was 47 years. The workers were aged 18 to 85 years. More than half of the workers had spent more than 15 years in the salt industry. The majority (85%) had heavy manual job tasks such as loading and unloading salt heaps, whereas 15% had moderate job task such as leveling the ground or constructing small mud walls for brine storage. There were no light-duty jobs. Less than half of the workers had completed primary school, and many were illiterate, particularly women (Table 1). Unlike women, who did not smoke or consume alcohol, half of the men smoked, and 2 out of 3 men consumed alcohol.

**Table 1:** Description of salt pan workers in Tamil Nadu, India

Variables	Men (*n* = 180)	Women (*n* = 172)	Total (*N* = 352)
Age^a^	46 (18–85)	45 (18–70)	46 (18–85)
Duration of salt pan work^a^	20 (1–70)	20 (1–45)	20 (1–70)
≥15 years of work in salt pans^b^	119 (66)	74 (43)	193 (54.8)
Litterate^b^	122 (68)	86 (50)	208 (59.1)
Smoker^b^	84 (47)	0	84 (23.8)
Use alcohol^b^	119 (66)	0	119 (33.8)
Morning weight^a^	53.9 (30.4–78.3)	49.9 (30.4–77.0)	53.9 (30.4–78.3)
BMI (Kg/m^2^)^a^	19.9 (17.1–31.9)	19.8 (17.2–34.5)	19.8 (17.1–34.5)
Glucosuria >1.6 mmol/l ^2^	4 (2)	4 (2)	8 (2.3)
Systolic BP mm Hg a	126 (96–200)	122 (80–220)	122 (80–220)
Diastolic BP mm Hg^a^	83.5 (54–120)	81 (59–120)	81 (54–120)
Heavy work^b^^,^^c^	162 (90)	135 (78)	297 (84.3)
High-heat stress (above TLV)^b^^,^^d^	169 (93.8)	139 (80.8)	308 (87.5)

BMI, body mass index; BP, blood pressure; TLV, threshold limit value.

a Median, range.

b N (%).

c Work category.^33^

d High workload above 27.5 **°**C WBGT, moderate work load above 28.0 **°**C WBGT.^33^

**Figure 2:**
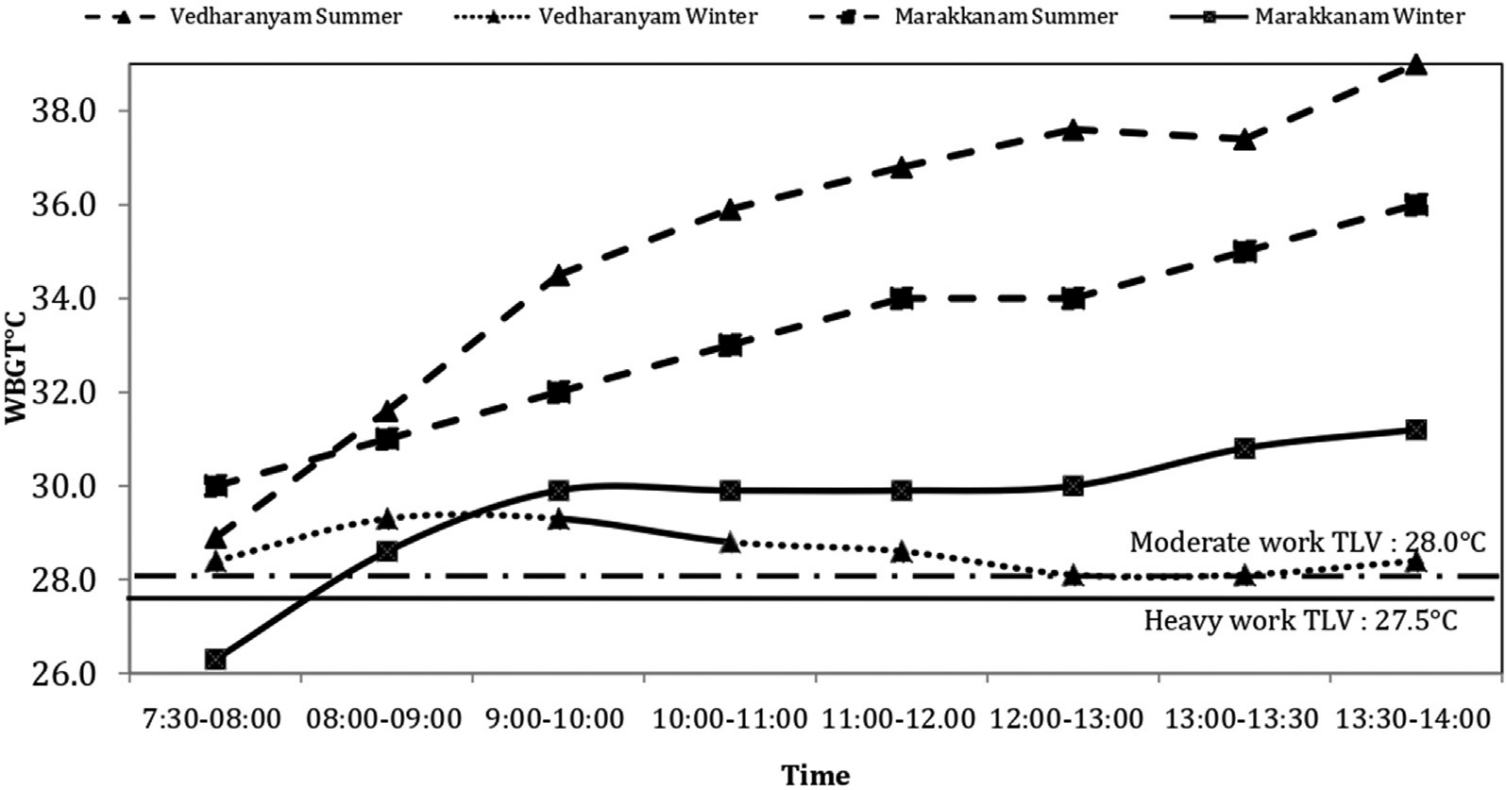
Average hourly Wet Bulb Globe Temperature measurements for a typical work shift in summer and winter seasons in 2 salt pans in Tamil Nadu, India. TLV, threshold limit value.

During the summer, continuous monitoring data from normal work shifts revealed that the WBGT exceeded the TLV as early as 7:30 am (Figure 2). In both seasons, WBGTs increased steadily throughout the workday, with the highest levels reported between 1:00 pm and 1:30 pm.

Workers in Marakkanam had a mean attributed WBGT of 31.1 ± 1.1 ˚C in the summer and 25.8 ± 1.6 ˚C in the winter, whereas those in Vedharanyam had a mean attributed WBGT of 30.3 ± 1.3 ˚C and 29.3 ± 1.1 ˚C in the summer and winter, respectively. Relative humidity, dry temperature, and WBGT are given in Supplementary Table S1. The prescribed TLV-WBGT limits, which consider workload, were surpassed in 95% of the 100 specific locations measured throughout the seasons, with the exception of 5 locations measured on cloudy days. Workers with a high workload had WBGT exposures that were always above the TLV.

### Self-Reported Symptoms, Physiological Changes Over the Workday, and Biomarkers of Renal Function

The majority of workers (93%) reported that they had experienced at least one of the self-reported heat-strain symptoms, here defined as excessive sweating, thirst, dizziness, muscle cramps, headache, nausea/vomiting, fainting, or prickly heat/rashes during the current season. The reported prevalence of each of these symptoms is given in Table 2. Dry mouth or severe thirst, all of which are signs of dehydration, were reported by 59% of the workers. Changes in urine volume or color, burning sensations when urinating, rashes, and urinary tract infections were widespread, with 77% of the workers reporting at least one of these symptoms. Workers bring their own 1-liter water bottle to sip throughout the day and have access to water for refilling bottles at work; however, the reported water consumption was low, and median was one Litre (Table 3). About 23% of women stated that if they had improved toilet access, they would drink more. One out of every 5 workers had taken sick leave owing to heat-related health difficulties in the last 6 months. Moreover, a third of the workers said they did not finish their work on time because they were working in the heat, resulting in missed income.

**Table 2:** Associations between heat stress and self-reported heat strain, dehydration symptoms, productivity losses, and physiological indicators of heat strain and kidney function in 352 salt pan workers from Tamil Nadu, India

Variables	Heat stress		Crude OR (95% CI)	AOR (95% CI)^c^
	Above TLV-WBGT limit^a^ (*n* = 308) %	Below TLV-WBGT limit^b^ (*n* = 44) %		
Heat strain symptoms				
Dizziness	49	16	5.0 (2.1–11.6)	7.3 (3.0–17.5)
Any dehydration symptom (Dry mouth, excessive thirst)	65	21	7.1 (3.2–15.3)	6.9 (3.1–15.1)
Tiredness/weakness	71	41	3.4 (1.8–6.5)	3.7 (1.9–7.4)
Excessive sweating	81	59	2.9 (1.5–5.6)	2.6 (1.3–5.2)
Nausea/Vomiting/ Fainting/Prickly heat	20	11	1.9 (0.7–5.1)	2.3 (0.8–6.4)
Thirst	75	64	1.7 (0.8–3.3)	1.4 (0.7–2.9)
Muscle cramps	53	52	1.1 (0.5–1.9)	1.1 (0.5–2.1)
Headache	43	48	0.8 (0.4–1.5)	1.1 (0.5–2.2)
Any heat strain symptom (any of above)	93	86	2.2 (0.8–5.6)	1.9 (0.7–5.2)
Urogenital symptoms				
Skin itching in urinogenital sites	17	9	1.9 (0.6–5.7)	1.7 (0.5–5.2)
Changes in urine volume/Color	78	68	1.7 (0.8–3.3)	1.6 (0.7–3.2)
Burning sensation during urination	26	18	1.6 (0.6–3.4)	1.4 (0.6–3.2)
Productivity losses				
Absenteeism/sick leave because of heat	23	14	1.9 (0.7–4.7)	2.0 (0.8–5.1)
Productivity loss/additional time to complete tasks	30	27	1.2 (0.5–2.3)	1.0 (0.5–2.2)
Wages lost because of heat	24	11	2.5 (0.9–6.6)	2.8 (1.0–7.5)
Physiological indicators of heat strain and kidney function				
Tympanic temperature, pre-post shift difference > 1 °C	14.3	18.2	0.7 (0.3–1.7)	0.6 (0.2–1.4)
SwR ≥1l/hr	52.6	18.2	4.9 (2.2–11.0)	4.2 (1.8–9.5)
Postshift USG ≥1.020	28.2	25.0	1.2 (0.5–2.4)	0.9 (0.4–2.1)
Postshift USG ≥1.025	8.8	4.5	2.1 (0.4–8.7)	1.7 (0.3–7.6)
eGFR <90 (ml/min per 1.73 m^2^)	51.3	22.7	3.5 (1.7–7.5)	2.9 (1.3–6.4)
eGFR <60 (ml/min per 1.73 m^2^)	6.8	4.5	1.5 (0.3–6.7)	0.9 (0.2–4.6)

AOR, adjusted odds ratios; CI, confidence interval; BMI, body mass index; BP, blood pressure; TLV, threshold limit value; USG, urine specific gravity; WBGT, wet bulb globe temperature.

a High work load above 27.5 **°**C WBGT, moderate work load above 28.0 **°**C WBGT.^33^

b Reference category.

c Adjusted for age (categorized as >40 years and <40 years) and gender.

**Table 3:** Physiological indicators measured over the workday in 352 salt pan workers from Tamil Nadu, India

Parameters	Pre-Shift median (range)	Post Shift median (range)	% of workers above cut-off pre shift	% of workers above cut-off post shift^a^	Difference between pre- and post shift^a^ *P*-Value^b^	Cut off (Reference range)
**Physiological heat strain indicators**						
Heart Rate (Beats/min)	80 (60–114)	85 (60–118)	2.2	4.2	<0.0001	<100^34^
Rise in Tympanic Temperature (^o^C)	36.5 (36–37.6)	37.0 (36–37.9)	-	14.8	<0.0001	<1°C Rise between pre and post^35^
Sweat Rate (l/hr)^c^	-	0.9; 0.1–2.1	-	48.3	-	<1(L/hr)^35^
Fluid intake (l)	-	1.0 (1.0–3.5)	-	-	-	-
**Dehydration indicators**						
pH	6.0; (5.0–8.0)	6.0; (5.0–8.0)	15.0	15.1	0.252	5–6^42^
Urine Specific Gravity (USG)	1.012; (1.002–1.030)	1.018; (1.002–1.030)	10.1	27.8	<0.0001	<1.020^38^
**Indicators of kidney function and injury**						
Leucocyturia(WBC/μl)	0 0–500	0 0–500	6.8	11.6	0.004	<10^43^
Hematuria (RBC/μl)	0 0–200	0 0–200	6.8	8.2	0.278	<5^43^
Proteinuria (mg/dl)	0 0–300	0 0–300	2.8	6.3	0.041	<2^43^
Serum creatinine (mg/dl)	-	0.9 (0.5–3.0)	-	10	-	Male: < 1.25 (Laboratory range)
	-	0.9 (0.4–2.4)	-	6.6	-	Female: <1.11 (Laboratory range)
Serum Uric acid (mg/dl)	-	3.1 (2.6–10.5)	-	2.3	-	Male: <7.2 (Laboratory range)
	-	3.05 (1.3–7.5)	-	1.7	-	Female: <6.0 (Laboratory range)
eGFR^d^ >90	-	105^e^ (90–139)	-	180 (51.1)^f^	-	National Kidney Foundation 2020^44^
eGFR^e^ 60-89	-	78^e^ (60–89)	-	146 (41.4)^f^	-	
eGFR^d^ <30-59	-	53^e^ (30–59)	-	25 (7.1)^f^	-	
eGFR^d^ <30	-	23^e^ (23–23)	-	1 (0.2)^f^	-	

eGFR, estimated glomerular filtration rate.

a The sampling was performed pre-shift and during the work day, mid- or post-shift.

b Wilcoxon paired signed test.

c Sweat Rate was calculated upon pre and post weight, fluid intake and duration of work hours.

d CKD-epi creatinine-based formula (ml/min/1.73 m^2^).

e Median (range) in category.

f N (%) in category.

Workers identified as having higher heat stress, or higher workload, more often reported more symptoms of dehydration than those with less demands (Table 2, Supplementary Table S2). Heat strain symptoms were also markedly more common among those with heavy workload. The crude point estimates did not change markedly with adjustments, neither for age, nor years of exposure.

The measured physiological responses are shown in Tables 2 and 3, as well as Supplementary Table S2 and Supplementary Figure S1. An HR of more than 100 beats per minute after the work shift was found in 4% of the workers. Tympanic temperature did not exceed 38 °C in any of the workers, and only in 15% of workers did it rise by more than 1 °C. Self-reported fluid intake during the workday was low, with median of 1 liter (range 0.25–3.5). The calculated SwR was greater than 1l/h in 48% of the workers, more often among workers with high-heat stress. Preshift and postshift USG, a proxy predictor for dehydration, was ≥1.020 in 10.1% and 28% of the workers, respectively. Urinary markers such as preshift and postshift leucocyturia were above the cutoff in 7% and 12% of workers postshift, respectively. Reduced eGFR (<90 ml/min per 1.73 m^2^) was seen in 56% (*n* = 101) of male workers and 38% (*n* = 66) of female workers, predominantly among workers with high-heat stress. eGFRs below 60 ml/min per 1.73 m^2^ were found in 7% of workers, of which 18 workers were males and 5 were females. The male workers had approximately 1.7-fold higher risk of eGFR (<90 ml/min per 1.73 m^2^) and approximately 3.1-fold higher risk of eGFR (<60 ml/min per 1.73 m^2^) compared to female workers after adjusting for years of exposure (>3 years and <3 years).

## Discussion

Salt pan workers were exposed to high levels of occupational heat stress from the start of the workday. All participants had heavy or moderate workload, and 9 out of 10 workers were estimated to work above the limits of heat exposure (WBGT-TLVs) when, according to international regulations, frequent break periods should be imposed.^33^ Such break periods were, however, not in place in any of the salt pans investigated.

Symptoms of dehydration were prevalent among workers, as were tiredness/weakness and other signs of heat strain. High USG levels observed before (10.1%) and after work (28%) and the average water intake of only 1 liter during the workday indicates a marked lack of rehydration. This likely depends not only on lack of access to palatable water during the workday but also indicates an adapted restricted fluid intake to avoid urination, a pattern which has been observed elsewhere in Indian settings without access to proper sanitation, especially among women.^10^ This exaggerates the risk of not being rehydrated while working in hot temperatures with excess sweating.

The strength of this salt pan study is the extensive WBGT monitoring, where variation in microclimate between locations and sampling days but limited variation within job tasks allowed the construction of job-exposure matrices for workload and heat stress based on WBGT-TLVs, which were then attributed to individual workers. The convenience sample can be seen as a limitation, but we have no reason to suspect that it was severely biased with respect to the outcomes of interest, being both self-reported and measured. The physiological measurements as well as the workload and heat stress classification represent a single day for the individual, but we presume that this day, at the group level, also is representative of labor over a longer period and thus work throughout a season, that is, the same time span as the questionnaire assessment of heat stress symptoms.

Measurement of HR at work in a hot climate reflects the combination of the external heat load, the workload, and the resulting internal heat generation. The participants were assessed a short while after leaving their actual job tasks, therefore with an expected lower HR than during ongoing work. The assessment of sweat rate was limited by self-reported fluid intake, and lack of measurement of urine voids during the observation period. Continuous HR measurements during the workday would have strengthened the assessment of the actual workload and heat stress, but neither this, nor closely monitored fluid balance, was feasible during this investigation. Still, we find that the simple and cheap methods used, which are available in other low-resource settings, gave valuable information.

Heat, frequent dehydration, and intense labor have been linked to an increased risk of nephrolithiasis in other occupations.^17^^,^^45^, ^46^, ^47^, ^48^ Here, 9% of the salt pan workers reported symptoms suggestive of kidney stones, which is less than among Indian steel workers with high-heat stress,^17^ and only a few had serum uric acid levels above the clinical reference range. The study had previously excluded hypertensives, but 23 workers had blood pressure above the 120/80 mmHg. This is in line with a high prevalence of hypertension in Indian disadvantaged populations.^49^

Almost half of the workers had S-Cr values corresponding to eGFR of less than 90 ml/min per 1.73 m^2^, and 7% had an eGFR of less than 60 ml/min per 1.73 m^2^, all without proteinuria. The venous samples were taken once during the workday. Ideally, venipuncture in the morning to assess baseline S-Cr for estimation of eGFR at steady-state, and again at the end of the workday to assess cross-shift elevation and acute kidney injury should be performed, but this was beyond the scope of the present study. The validity of a creatinine-based Chronic Kidney Disease Epidemiology Collaboration equation in this disadvantaged Indian community may be called into question because a recent study from northern India found that all creatinine-based equations significantly overestimated glomerular filtration, whereas cystatin C equations were more accurate.^50^ As a result, the true prevalence of decreased glomerular filtration in this cohort of vulnerable workers is likely to be underestimated. There are indeed reasons for extended investigations of kidney health among salt pan workers, in a first step as cross-shift and cross-harvest investigations.

In these heat-exposed manual workers, the finding of reduced eGFR in the absence of proteinuria and diabetes is similar to what has been reported in Mesoamerican sugarcane and construction workers.^18^ Our findings are also in accordance with findings in Thai salt pan workers,^31^ Chronic exposures to heat, paired with strenuous activity that creates metabolic heat and insufficient rehydration to compensate for sweat losses, are risk factors for kidney injury, according to mounting evidence.^3^^,^^21^^,^^51^^,^^52^ A single spot SCr test is not diagnostic for renal disease at the individual level, but group level findings of impaired kidney function in the absence of diabetes and hypertension in cross-sectional research is indicative of an increased presence of kidney disease in the population.

The reported average intake of only 1 liter of water per day in bottles brought from home clearly speaks for workplace interventions to provide palatable water, safe sanitation, and education on the need to hydrate. Low technology workplace implementations focusing on regulated rests in shade, access to water and sanitation, and an understanding of why this is needed among employers and workers have been shown to reduce heat stress and kidney function loss in industrial agriculture,^48^^,^^53^, ^54^ and should be equally effective in the salt pans.

### Conclusion

Our study performed with cheap and commonly available methods highlights the presence of high-heat exposure, workload, and physiological heat strain among these nonagricultural outdoor workers. In addition, a high prevalence of decreased kidney function was indicated. In a changing climate scenario, the expected rise in temperature will increase heat exposure and have negative health consequences for a large global workforce. Heat-exposed outdoor workers, especially from poorer areas, are among the most vulnerable populations. Even small increases in global temperature, as predicted by climate change,^1^ will have a significant impact on the workforce in India and other tropical countries where workers are already exposed to dangerously high temperatures. Although continuing research in the salt pans using a seasonal approach will allow temporal trends to be discovered and kidney health effects better described, it is obvious that sustainable workplace interventions with a comprehensive approach are urgently needed. The steps to building safe work practices include monitoring exposures and heat-related diseases, reducing heat stress, and enhancing workers' hydration and sanitation.

## Disclosure

All the authors declared no competing interests.

## Supplementary Material

Supplementary File (PDF)

**Figure S1.** Cross-shift changes in the physiological changes among 352 salt pan workers from Tamil Nadu, India.

**Table S1.** Attributed dry bulb temperature, relative humidity, and Wet Bulb Globe Temperature in 352 salt pan workers in Tamil Nadu, India.

**Table S2.** The associations between workload and self-reported heat strain dehydration symptoms, productivity losses, and physiological indicators of heat strain and kidney function in 352 salt pan workers from Tamil Nadu, India.
