# Supplementary material for: Occupational Heat Stress and Kidney Health in Salt Pan Workers
Source: Kidney Int Rep. 2023 Apr 20;8(7):1363–72. doi: 10.1016/j.ekir.2023.04.011 (PMC10334398; doi:10.1016/j.ekir.2023.04.011)
Supplement: Supplementary File (PDF) [file mmc1.pdf]

Supplementary Materials:

Table S1: Attributed dry bulb temperature, relative humidity and Wet Bulb Globe Temperature (WBGT) in 352 saltpan workers in Tamil Nadu, India

Area	**Season (partici-pants)**	**Dry Bulb* (°C)**			**Relative Humidity* (%)**			**WBGT (°C)**		
		**Min**	**Max**	**Mean ±SD**	**Min**	**Max**	**Mean ±SD**	**Min**	**Max**	**Mean ±SD**
Marakkanam	Summer (N=53)	**30.7**	**35.9**	33.6±1.2	**38**	**77**	52.2±8.7	28.5	32.8	31.1±1.1
	Winter (N=42)	**21.9**	**29.2**	25.3±2.3	**52**	**90**	66.8±10.1	22.6	28.3	25.8±1.9
Vedharanyam	Summer (N=201)	**29.8**	**36**	32.2±1.3	**38**	**80**	58.1±8.8	26.5	33.3	30.3±1.3
	Winter (N=56)	**29.8**	**36**	32.8±1.4	**39**	**66**	50.6±6.2	26.5	33.3	29.3±1.3

** Average ambient parameters during the workplace heat measurements.*

Table S2: The associations between workload and self-reported heat strain dehydration symptoms, productivity losses, and physiological indicators of heat strain and kidney function in 352 saltpan workers from Tamil Nadu, India

	Work load[1]		Crude OR (95% CI)	AOR (95% CI)[2]
	**Moderate workload[1] % (n=55)**	**Heavy workload[1] % (n=297)**		
**Heat strain symptoms**				
Dizziness	10.9	50.8	**8.4 (3.5-20.3)**	**8.1 (3.3-19.8)**
Nausea/Vomiting/ Fainting/ Prickly heat	3.6	21.9	**7.4 (1.7-31.2)**	**7.5 (1.7-32.1)**
Tiredness/weakness	29.1	73.7	**6.8 (3.6-12.9)**	**6.8 (3.5-13.1)**
Excessive sweating	47.3	83.8	**6.4 (3.3-12.2)**	**6.3 (3.3-12.1)**
Any dehydration symptom (Dry mouth, excessive thirst)	23.6	65.7	**6.2 (3.1-12)**	**6.3 (3.1-12.4)**
Any heat strain symptom (any of above)	80.0	94.6	**4.4 (1.9-10)**	**3.5 (1.5-8.9)**
Thirst	50.9	77.8	**3.3 (1.8-6.1)**	**3.2 (1.7-6.1)**
Muscle cramps	32.7	56.9	**2.7 (1.4-4.9)**	**2.4 (1.3-4.6)**

Headache	30.9	46.1	**1.9 (1.1-3.5)**	**2.0 (1.1-3.8)**
**Urogenital symptoms**				
Changes in urine volume/Color	47.3	82.5	**5.2 (2.8-9.6)**	**5.5 (2.9-10.5)**
Burning sensation during urination	12.7	26.9	2.5 (1.1-5.8)	2.6 (1.1-6.1)
Skin itching in urinogenital sites	7.3	17.2	2.6 (0.9-7.6)	2.4 (0.8-7.1)
**Productivity losses**				
Productivity loss/additional time to complete tasks	**14.5**	**32.7**	**2.8 (1.2-6.2)**	**3.6 (1.6-8.2)**
Wages lost due to heat	**9.1**	25.3	**3.3 (1.2-8.7)**	**3.2 (1.2-8.5)**
Absenteeism/ sick leave due to heat	**10.9**	**24.2**	**2.6 (1.1-6.3)**	**2.5 (1.1-6.2)**
**Physiological indicators of heat strain and kidney function**				
[9]SwR ≥1L/hr	**25.5**	**52.5**	**3.2 (1.7-6.1)**	**3.8 (1.9-7.6)**
Tympanic temperature pre-post difference > 1°C	**12.7**	**15.2**	1.2 (0.5-2.8)	1.1 (0.4-2.6)
eGFR<90	**38.2**	**49.5**	1.6	1.4

(mL/min/1.73 m^2)			(0.8-2.8)	(0.7-2.6)
Post USG ≥1.025	**7.2**	**8.4**	1.2 (0.3-3.5)	1.1 (0.3-3.3)
Post USG ≥1.020	**29.1**	**27.8**	0.9 (0.4-1.7)	0.9 (0.4-1.7)
eGFR<60 (mL/min/1.73 m^2)	**7.3**	**6.4**	0.9 (0.3-2.9)	0.8 (0.2-2.6)

[1]Assessed according to ACGIH (2018)

[2]Adjusted for age (categorised) and gender

Table S3: STROBE Statement—checklist of items for observational studies

	Item No	Recommendation	Remarks
**Title and abstract**	1	(*a*) Indicate the study's design with a commonly used term in the title or the abstract	Added
		(*b*) Provide in the abstract an informative and balanced summary of what was done and what was found	Added
**Introduction**			
Background/rationale	2	Explain the scientific background and rationale for the investigation being reported	Added
Objectives	3	State specific objectives, including any pre-specified hypotheses	Added
**Methods**			
Study design	4	Present key elements of study design early in the paper	Added
Setting	5	Describe the setting, locations, and relevant dates, including periods of recruitment, exposure, follow-up, and data collection	Added
Participants	6	(*a*) *Cohort study*—Give the eligibility criteria, and the sources and methods ofselection of participants. Describe methods of follow-up *Case-control study*—Give the eligibility criteria, and the sources and methods of case ascertainment and control selection. Give the rationale for the choice of casesand controls	Added

		Cross-sectional study—Give the eligibility criteria, and the sources and methods of selection of participants	
		(*b*) *Cohort study*—For matched studies, give matching criteria and number ofexposed and unexposed<br>*Case-control study*—For matched studies, give matching criteria and the number of controls per case	
Variables	7	Clearly define all outcomes, exposures, predictors, potential confounders, and effect modifiers. Give diagnostic criteria, if applicable	Added
Data sources/ measure ment	8 *	For each variable of interest, give sources of data and details of methods of assessment (measurement). Describe comparability of assessment methods if there is more than one group	Added
Bias	9	Describe any efforts to address potential sources of bias	Added
Study size	1 0	Explain how the study size was arrived at	Added
Quantitative variables	1 1	Explain how quantitative variables were handled in the analyses. If applicable,<br>describe which groupings were chosen and why	Added
Statistical methods	1 2	(*a*) Describe all statistical methods, including those used to control for confounding	Added
		(*b*) Describe any methods used to examine subgroups and interactions	Added
		(*c*) Explain how missing data were addressed	
		(*d*) *Cohort study*—If applicable, explain how loss to follow-up was addressed<br>*Case-control study*—If applicable, explain how matching of cases and controls wasaddressed<br>*Cross-sectional study*—If applicable, describe analytical methods taking account of<br>sampling strategy	Added
		(*e*) Describe any sensitivity analyses	
Continued on next page			
**Results**			
Participants	13*	(a) Report numbers of individuals at each stage of study—eg numbers potentially eligible, examined for eligibility, confirmed eligible, included in the study, completing follow-up, and analysed	Added

		(b) Give reasons for non-participation at each stage	
		(c) Consider use of a flow diagram	
Descriptive data	14*	(a) Give characteristics of study participants (eg demographic, clinical, social) and information on exposures and potential confounders	Added
		(b) Indicate number of participants with missing data for each variable of interest	Added
		(c) *Cohort study*—Summarise follow-up time (eg, average and total amount)	
Outcome data	15*	*Cohort study*—Report numbers of outcome events or summary measures over time	
		Case-control study—Report numbers in each exposure category, or summary measures of exposure	
		Cross-sectional study—Report numbers of outcome events or summary measures	Added
Main results	16	(*a*) Give unadjusted estimates and, if applicable, confounder-adjusted estimates and their precision (eg, 95% confidence interval). Make clear which confounders were adjusted for and why they were included	
		(*b*) Report category boundaries when continuous variables were categorized	Added
		(*c*) If relevant, consider translating estimates of relative risk into absolute risk for a meaningful time period	
Other analyses	17	Report other analyses done—eg analyses of subgroups and interactions, and sensitivity analyses	Added
**Discussion**			
Key results	18	Summarise key results with reference to study objectives	Added
Limitations	19	Discuss limitations of the study, taking into account sources of potential bias or imprecision. Discuss both direction and magnitude of any potential bias	Added
Interpretation	20	Give a cautious overall interpretation of results considering objectives, limitations, multiplicity of analyses, results from similar studies, and other relevant evidence	Added
Generalisability	21	Discuss the generalisability (external validity) of the study results	Added
**Other information**			
Funding	22	Give the source of funding and the role of the funders for the present study and, if applicable,for the original study on which the present article is based	Added

*Give information separately for cases and controls in case-control studies and, if applicable, for exposed andunexposed groups in cohort and cross-sectional studies.<br><br>**Note:** An Explanation and Elaboration article discusses each checklist item and gives methodological background and published examples of transparent reporting. The STROBE checklist is best used in conjunction with this article (freelyavailable on the Web sites of PLoS Medicine at http://www.plosmedicine.org/, Annals of Internal Medicine at http://www.annals.org/, and Epidemiology at http://www.epidem.com/). Information on the STROBE Initiative is available at www.strobe-statement.org.	

Core Body Temperature

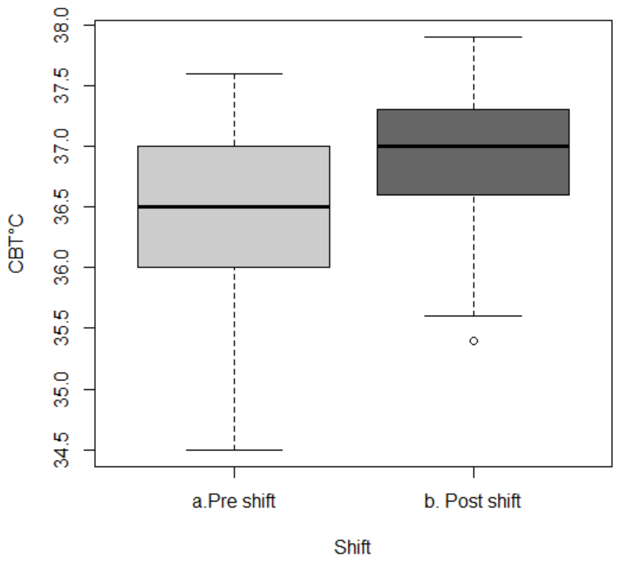

Urine Specific Gravity

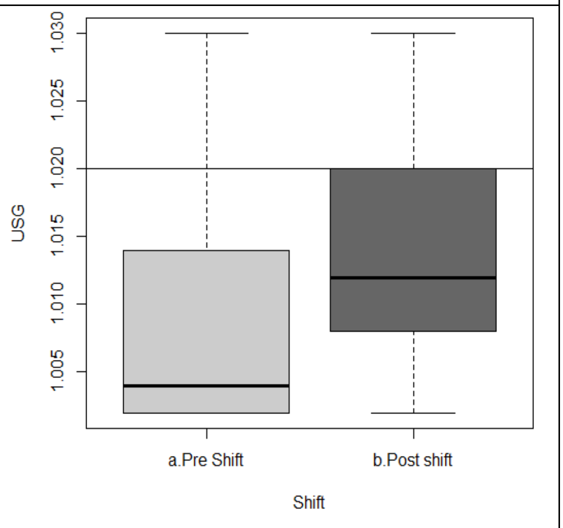

Figure S3: Cross shift changes in the physiological changes among 352 saltpan workers
